# Case report: Epidural blood patches are effective in treating intracranial hypotension due to a subarachnoid-pleural fistula

**DOI:** 10.3389/fsurg.2022.936949

**Published:** 2022-09-27

**Authors:** Hua Huang, Ting-Ting Wei, Zhong-Feng Niu, Liang Yu, Fei-Fang He

**Affiliations:** ^1^ Department of Pain Management, Center for Intracranial Hypotension Management, Sir Run Run Shaw Hospital, School of Medicine, Zhejiang University, Hangzhou, China; ^2^ Department of Anesthesiology, Sir Run Run Shaw Hospital, School of Medicine, Zhejiang University, Hangzhou, China; ^3^ Department of Radiology, Sir Run Run Shaw Hospital, School of Medicine, Zhejiang University, Hangzhou, China; ^4^ Department of Quality Management, Sir Run Run Shaw Hospital, School of Medicine, Zhejiang University, Hangzhou, China

**Keywords:** posterior mediastinal mass, Tarlov cyst, subarachnoid-pleural fistula, intracranial hypotension, epidural blood batch

## Abstract

**Background:**

Intracranial hypotension (IH) is usually associated with cerebrospinal fluid (CSF) leakage and/or CSF hypotension, and epidural blood patch (EBP) therapy has been proven to be effective for treating spontaneous IH and post-dural puncture headaches. Tarlov cysts (TCs) are common lesions of the sacral spine. They have rarely been reported in thoracic locations and are even less common in the posterior mediastinum, which can lead to their misdiagnosis as neurogenic tumors.

**Case presentation:**

Here, we report the case of a 60-year-old woman who developed an orthostatic headache after the thoracoscopic resection of a TC in the posterior mediastinum that was presumed to be a schwannoma preoperatively. The patient was finally diagnosed with IH caused by a subarachnoid-pleural fistula (SPF) and was cured by targeted EBP treatment.

**Conclusion:**

This is a case to show that a single targeted EBP treatment is effective for a patient with IH caused by an SPF after thoracoscopic resection of a TC. This case reminds us to be vigilant that a TC may be masquerading as a posterior mediastinal neurogenic tumor, and a detailed examination should be performed to identify it before deciding on a surgical procedure. In addition, postural headache after thoracoscopic spinal surgery should be alert to the possibility of IH induced by an SPF. Once it occurs, early treatment is necessary, and targeted EBP treatment can serve as a safe and effective alternative when conservative treatment fails.

## Introduction

Intracranial hypotension (IH) is a disorder that causes cerebrospinal fluid (CSF) hypovolemia, and it is usually due to spontaneous or traumatic CSF leakage. IH is typically characterized by the presence of an orthostatic headache, and associated symptoms are common, including nausea, vomiting, neck stiffness, hearing changes, diplopia, visual field damage, cognitive abnormalities, coma and even death in rare cases (1–4). Epidural blood patch (EBP) treatment is usually considered in the management of moderate to severe headaches attributed to low CSF pressure that fails to respond to conservative management, such as post-dural puncture headaches, CSF fistula headaches, and spontaneous IH headaches (5).

A Tarlov cyst (TC) is defined as a CSF-filled cystic lesion in the epidural space that forms in the nerve root sheath of the dorsal root ganglion. Thoracic TCs are very uncommon and to the best of our knowledge, there was only one reported case of a thoracic TC located in the posterior mediastinum (6). Therefore, this was the second reported case of a TC in the posterior mediastinum, making it difficult to distinguish it from a neurogenic tumor. TCs are suggested as a possible cause of IH in so-called idiopathic cases (7). In addition, IH due to CSF leakage is one of the most common complications after surgical treatment for TCs, and these surgical treatments include microsurgical cyst fenestration and imbrication, partial cyst resection, cyst resection and cystectomy (misdiagnosed as pelvic masses) (8–10). This is the first reported case of a patient who suffered from IH due to a subarachnoid-pleural fistula (SPF) after thoracoscopic resection of a TC that was misdiagnosed as a schwannoma, and this patient was finally cured by targeted EBP treatment.

## Case description

**Figure 1:**
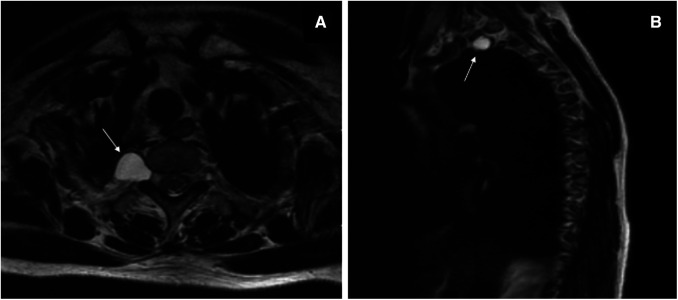
T2-weighted magnetic resonance imaging before the thoracoscopic surgery. Axial image (**A**) and sagittal image (**B**) showing a hyperintense oval lesion, with signal intensity similar to fluid, originating in the posterior mediastinum, protruding into the apical thorax at the level of the right T1–T2 intervertebral foramen (*white arrows*).

**Figure 2:**
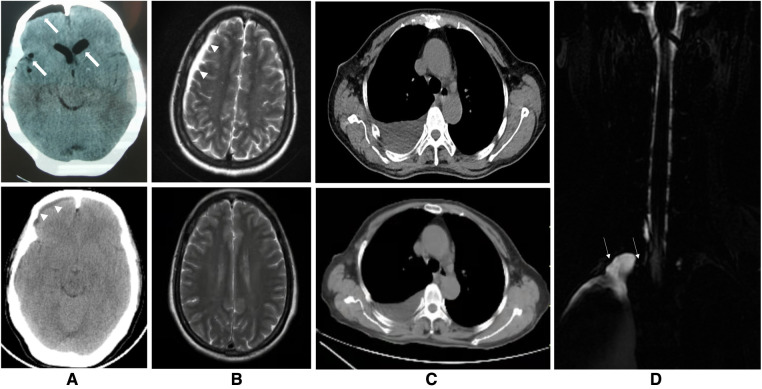
Images of the brain, chest and spine. (**A**) The upper figure of the axial computed tomography (CT) showing pneumocephalus in the cranial cavity of the lateral ventricle and right frontal, parietal and temporal and ventricular dilatation 5 days after the thoracoscopic surgery (*thick arrows*) and the lower figure showing resolution of these findings and presence of right frontal subdural effusion 25 days after the thoracoscopic surgery (*arrowheads*). (**B**) The upper figure of axial T2-weighted magnetic resonance imaging of the brain showing subdural hematomas in the left frontal region, right frontotemporal parietal occipital region before the epidural blood patch (EBP) therapy (*arrowheads*) and the lower figure showing complete absorption of these subdural hematomas 2 months after EBP therapy. (**C**) The upper figure of axial chest CT demonstrating a moderate amount of right-sided pleural effusion before the EBP therapy and the lower figure showing decrease of it 2 months after EBP therapy. (**D**) T2-weighted magnetic resonance myelography imaging of the spine showing subarachnoid-pleural fistula connection of the left pleural effusions and the spinal canal before EBP therapy (*white arrow*).

A 60-year-old woman was admitted to the Department of Thoracic Surgery in a local hospital with a history of dry cough and vague upper back pain for 1 year. General examination showed no significant findings. Chest computed tomography (CT) showed there was a posterior mediastinal mass on the right side. Magnetic resonance imaging (MRI) revealed that the lesion (measuring 1.4 cm × 1.9 cm × 1.5 cm) was located in the thoracic apical space on the right side of the T1–T2 intervertebral foramen. The lesion was hypointense on T1-weighted MRI and hyperintense on T2-weighted MRI (Figure 1). The lesion was thought to be a schwannoma and a complete surgical resection of the lesion under thoracoscopy was then performed. However, the postoperative pathological findings showed the presence of fibrous tissue with nerve tissue, which is typical for TCs. On day 1 postoperatively, the patient vomited and developed a severe burst headache. The headache was mainly localized on the top of the head and around the occipital area. The headache was orthostatic, occurring within 30 s of the patient assuming an upright position, was relieved after several minutes of lying down, and was occasionally accompanied by bilateral tinnitus. The pain was almost constant, was sometimes of high intensity (8/10 on a verbal numeric scale). She was afebrile, and the results of a neurological examination showed no obvious abnormalities. The patient was suspected of having IH and received conservative treatment, such as absolute bed rest and iv rehydration. On day 5 postoperatively, a brain CT was performed, and it showed intracranial pneumatosis (Figure 2A) and she was discharged after her symptoms improved. However, 25 days after the operation, the patient again developed a severe orthostatic headache after sudden vomiting. Back to the local hospital, a brain CT showed the absorption of intracranial pneumatosis and presence of right frontal subdural effusion, a further MRI examination of the brain showed that the patient had bilateral subdural hematomas (SDHs), and a chest CT showed a moderate amount of right-sided pleural effusion (Figures 2A–C). However, this time, after conservative treatment, the patient's symptoms did not improve, and the patient was transferred to the Department of Pain Management in our hospital.

**Figure 3:**
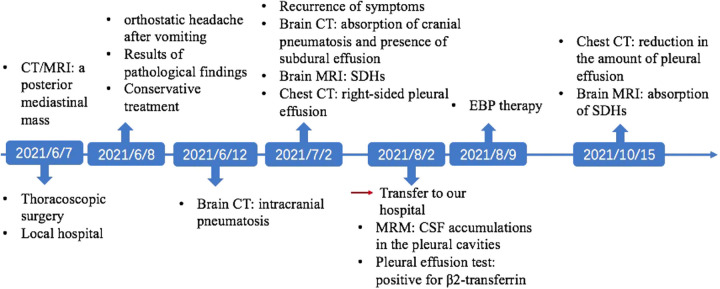
Timeline of the patient's clinical courses.

Magnetic resonance myelography (MRM) imaging of the spine obtained after admission to our hospital showed CSF accumulations in the pleural cavities over the right lung and T2-weighted MRI imaging of the entire spine showed no abnormal results within the operative areas (Figure 2D). The screenings for cancerous and immunological disorders revealed no abnormalities. Her clinical and radiographic findings supported a provisional diagnosis of symptomatic IH secondary to an SPF. Therefore, we performed an analysis on the pleural effusion and found that it was positive for β2-transferrin, which is specific to CSF, confirming the presence of an SPF. Therefore, a final diagnosis of SPF-induced IH that was complicated with SDHs was made. Given the failure of conservative treatment and the patient's stable condition, a targeted EBP treatment was administered 63 days after the first surgery. The procedure was performed using biplanar fluoroscopy with the patient in the prone position with a pillow under the patient's chest. An 18-gauge RX-2 Coude® epidural needle was slowly inserted into her epidural space at the T1/2 level using the median approach under fluoroscopic guidance. After confirming that the needle was in the epidural space after the visualization of the contrast agent in the target site in the lateral and anteroposterior fluoroscopic views, 14 ml of autologous peripheral blood that had been collected from the left brachial vein was infused. As a precautionary measure, the patient remained in the prone position for 1 h after the procedure and then was moved to the supine position with strict bed rest for the next 3 days. Four days after EBP therapy, the patient's headache was markedly relieved, and her clinical picture got complete resolution within 3 weeks. Two months after EBP therapy, a follow-up brain MRI showed bilateral SDHs size reduction with complete disappearance, and a chest CT showed a marked reduction in the amount of pleural effusion (Figures 2B,C and timeline in Figure 3). Over a 6-month follow-up period, none of her symptoms recurred, which suggests a favorable recovery.

## Discussion

A TC refers to a cystic dilatation that occurs between the endoneurium and perineurium of the nerve roots, and these cysts are filled with CSF (11). TCs are most often found in the lumbosacral spine, with a prevalence of 1.5% to 4.6%, and 80% of them are asymptomatic (12). Thoracic TCs are very uncommon, and accounted for 5.5% of all the cysts detected in one retrospective study (13). MRI is the most useful modality to detect TCs, with typical imaging showing a signal intensity that is consistent with cysts filled with CSF. However, the final diagnosis is made histologically, which is characterized by the presence of nerve fibers within the fibrous tissue of the cyst wall (14). Thoracic TCs are very uncommon, and there has only been one reported case of a thoracic TC located in the posterior mediastinum. In this case, the rarity of TC in the posterior mediastinum and its similar appearances on noncontrast MRI with a schwannoma resulted in an initial misdiagnosis. Although there is a call for less contrast-enhanced examinations due to the unknown toxicity of gadolinium in body tissue and its extra cost of time and money, we still recommended a gadolinium-enhanced T1-weighted MRI for this condition, as the gadolinium-enhanced T1-weighted MRI can show a low intensity area for a TC but a high intensity area for the solid component of a schwannoma.

Currently, the treatment for TCs remains controversial. Expectant therapy is generally recommended for most asymptomatic TC patients (15). When the patient is symptomatic, the published treatment options include cyst aspiration, the perineural injection of a glucocorticoid, the injection of blood or fibrin sealant into the cyst, and surgical treatment, which usually involves a small laminectomy with cyst wall fenestration with or without packing the remnant space with fat graft or excision while avoiding neural tissues (16). In this case, a TC was located in the posterior mediastinum at the T1–T2 level, of which there has only been one previously reported case; the symptoms of dry cough and vague upper back pain led to the patient's consultation with a thoracic surgeon and ultimately to the misdiagnosis of a schwannoma. Thoracoscopic complete resection was then performed, as this technique has been proven to be feasible and safe for the management of small mediastinal neurogenic tumors that are in apical locations (17, 18). However, the postoperative pathologic findings showed fibrous tissue, along with nerve tissue, which is typical for TCs. Due to the use of thoracoscopic surgery, it is difficult to avoid intraoperative dural damage, and it can result in a rare iatrogenic SPF.

An SPF is an abnormal communication between the subarachnoid space and pleural cavity, resulting in uncontrolled CSF drainage. In this case, an iatrogenic SPF was caused by injury of the dural sleeve during thoracoscopic surgery after the misdiagnosis of a TC preoperatively; it is likely that the dural injury was concealed, with no obvious outflow of CSF during the surgery. It is important to note that CSF leakage can also occur when a definite diagnosis of TC has been made in advance, as CSF leakage is reported to be the most frequent complication of surgical interventions for TCs, as shown in a systematic review (19). This may be because an intercostal nerve dural sleeve tear is easily overlooked, as it can be obscured by fat or peridural venous oozing at the level of the neural foramen (4). After a root avulsion, the CSF outflow causes cerebral collapse with arachnoid mater tearing and air entry into the intracranial subdural space through defects due to thoracotomy, leading to intracranial pneumatosis (20). This is what we observed on this patient's cranial CT, which was performed on day 5 postoperatively. The SDHs that were detected on day 25 postoperatively in this patient were believed to have resulted from a bridging vein rupture as the CSF volume had decreased (21). Although our patient presented with no focal neurological dysfunction other than a headache, the severity of her symptoms, the imaging findings, and the awareness of the SPF and its potential for related morbidity and even mortality supported aggressive intervention once conservative management failed.

Although only a handful of cases have been reported, treatments for SPF include conservative treatments (i.e., chest drainage, bed rest, intravenous fluid supplementation, noninvasive positive pressure ventilation, etc.), and surgical repair (22, 23). However, direct surgical repair of the incidental dural tear is considered both technically demanding and time-consuming due to the anatomical and surgical constraints of the thorax, including negative intrathoracic pressures, constrained working channels and limited visualization (24).

Due to the failure of conservative treatment and although this patient was complicated with SDHs, which are serious complications of IH, the SDHs in this patient were small in size with no obvious cerebral compression, and the patient was in a stable condition. Therefore, due to the limitations of open surgery, we decided to give priority to targeted EBP treatment. There is growing evidence on the efficacy of EBP in the management of SPF. Sheng-Feng Lin et al. reported a similar case of a patient with IH caused by an SPF after chiropractic manipulation, and this patient was successfully cured by repeated EBP therapies (25). EBP therapy, as a supplementary measure to address SPF, has been tried based on the belief that, the injected blood reduces the volume of the spinal canal, thus limiting the impact of CSF hypovolemia and relieving the patients' symptoms (26). Second, the blood may also provide an additional benefit by sealing the dural leak with a blood clot that can be tightly adhered to the dural (27).

In this case, we administered treatment with a targeted EBP rather than a blind EBP for several reasons. First, although both blind and targeted EBP treatment have been shown to be effective in treating IH, it has been supported in several studies that targeted EBP treatment may be more effective than blind EBP treatment (28–31). Second, CT or MRM is required to determine the location and extent of CSF leakage before administering the targeted EBP treatment; however, CT myelography is associated with a radiation risk and other risks, such as intrathecal puncture, chemical meningitis, spinal cord and nerve root compression, and seizures (29). In this case, MRM was used to detect the CSF leakage site, because MRM is advantageous in that it is noninvasive and has no risk of radiation exposure. Third, it has been reported that most EBPs extend only three to five segments from the injection sites, we believed that conventional treatment with a blind EBP *via* the lumbar approach may be insufficient in covering this patient's thoracic defect (27). Furthermore, we have noticed that another patient was reported to have undergone two failed EBP treatments after experiencing iatrogenic IH and a “sagging brain” due to a persistent SPF after a thoracotomy and a lobectomy for lung carcinoma, and this patient was finally cured through surgical repair (22). We hypothesized that the failure of EBP treatment in that patient may be caused by the failure of blind EBP treatment to accurately locate the dural defect or to cover the large dural defect. Therefore, due to the limitation of the small sample size of cases in the literature, we may need more evidence to clarify the effectiveness of targeted EBP therapy for patients with IH caused by an SPF and to further define the details regarding the severity of dural tears and proper treatment plans. In our patient, the clinical symptoms were rapidly alleviated, the SDHs were absorbed, the pleural effusion decreased, and the symptoms did not recur, all suggesting that the targeted EBP treatment was effective and safe. Therefore, currently, we recommend targeted EBP treatment as an alternative after the failure of conservative treatment in those patients with IH caused by an SPF, but strict monitoring and follow-up should be performed.

## Conclusion

An SPF can be difficult to treat if the patient fails conservative treatment, and it can be a potential source of mortality and significant morbidity. Here, our case report presents a patient with uncommon imaging findings of a TC, and this case highlights the significance of a thorough examination of a posterior mediastinal mass with an unclear diagnosis before deciding on the operation to prevent the occurrence of an SPF. In addition, this case demonstrates that targeted EBP therapy is effective and safe for patients who have IH caused by an SPF when conservative treatment fails but the patient is in a stable condition.

## Data Availability

The original contributions presented in the study are included in the article/Supplementary Material, further inquiries can be directed to the corresponding author/s.

## Author’s note

The authors have read the CARE Checklist (2016), and the manuscript was prepared and revised according to the CARE Checklist (2016).
